# An anatomical classification of congenital proximal radioulnar synostosis based on retrospective MRI measurement combined with radiography

**DOI:** 10.1038/s41598-022-09411-6

**Published:** 2022-04-21

**Authors:** Jin Li, Kailei Chen, Jing Wang, Yueming Guo, Saroj Rai, Xin Tang, Chinese Multi-center Pediatric Orthopedic Study Group (CMPOS), Jin Li, Xin Tang, Yueming Guo

**Affiliations:** 1 Department of Orthopedics, Union Hospital, Tongji Medical College, Huazhong University of Science and Technology, Wuhan, 430022 China; 2 Tongji Medical College, Huazhong University of Science and Technology, Wuhan, 430030 China; 3 Department of Radiology, Union Hospital, Tongji Medical College, Huazhong University of Science and Technology, Wuhan, 430022 China; 4 Department of Pediatric Orthopedics, Foshan Hospital of Traditional Chinese Medicine, Foshan, 528000 China; 5 Department of Orthopaedics and Trauma Surgery, Blue Cross Hospital, Tripureswor, Kathmandu 44600 Nepal

**Keywords:** Musculoskeletal system, Paediatric research

## Abstract

Existed classifications of congenital proximal radioulnar synostosis (PRUS) mainly focus on osseous changes and do not cover all types of congenital PRUS, ignoring the role and developing status of the supinator. This study aims to explore the correlation between supinator development and radiographic deformity of congenital PRUS. Pediatric patients diagnosed with congenital PRUS in two pediatric Orthopedic centers were evaluated retrospectively. MRI and radiographic images of their bilateral forearms (including normal ones) were collected. The area of supinator, extensor carpi radialis longus (ECRL), extensor carpi radialis brevis (ECRB), brachioradialis (BRAR) muscle and extensor indicis (EI) muscle were measured on each forearm. The ratios of these muscles were calculated and regarded as an indicator of the developing status of supinator muscle. Twenty-seven congenital PRUS forearms of 16 patients (average 3.45 years) were included. A new MRI & X-ray classification system was proposed to cover all types of radiographic deformity and provide a comprehensive description of supinator development. This study revealed the relation between MRI measured supinator volume and radiographic deformity of congenital PRUS. Supinator muscles were observed in all congenital PRUS cases. A novel classification was proposed, providing a more comprehensive understanding of congenital PRUS.

## Introduction

Congenital proximal radioulnar synostosis (PRUS) is a rare hereditary disease transmitted as an autosomal dominant pattern and most common congenital malformation around the elbow joint^1^. Sandifort initially described congenital PRUS in Museum Anatomicus in 1793^1–3^. The disease is characterized by difficulty in forearm supination and elbow extension^4^. The supination is compensated by the external rotation of the wrist and shoulder joint. The patient has only a slight cosmetic abnormality if the shoulder and wrist movements offer enough compensation^5^. However, some patients might show severe pronation deformity and have difficulty in daily activities such as washing face, dressing and using chopsticks or other eating utensils^2,6^. Congenital PRUS is more common in males than females^7–10^. Bilateral involvement occurs in 60% ~ 80% of cases^11^. However, no specific treatment modality has been described yet^9,12–14^. Surgeons usually rely on osseous changes on X-ray and three dimensional (3D) computed tomography (CT) for surgical treatment^15,16^. Available classification systems such as Cleary & Omer and Tachdjian classification also mainly discuss on radiographic abnormality^17,18^. However, they failed to comprehensively cover all reported congenital PRUS deformities and ignored changes in forearm muscles and their kinematics. The correlation between forearm muscles and radiographic deformity is still unknown. Consequently, neither development nor volume of forearm muscles was considered when choosing treatment protocol and predicting prognosis.

Supinator is a deep muscle located at the proximal end of the forearm, covered by extensor carpi radialis longus, extensor carpi radialis brevis, brachioradialis muscle and extensor indicis muscle. It is one of the primary muscles responsible for forearm supination^19,20^. The reason for supinator muscle atrophy, fibrosis or absence might lead to dysfunctions^8,14^, resulting in forearm pronation that directly affects the activities of daily living (ADL)^10,21^. Published studies on congenital PRUS mainly discussed the correlation between osseous abnormality and recovery process through X-ray or 3D CT analysis^16^. The function and volume of forearm muscles are also crucial for surgical planning and prognosis prediction. But there is no appropriate measurement method. Electromyography is widely applied in measuring muscular function. But the activation of deep-located muscle is not easy to precisely measure by the electrodes on the skin^22^. The muscular volume can be evaluated by imaging techniques. However, the CT scan does not provide enough contrast for muscle, adipose and connective tissue. Similarly, ultrasound does not have a large enough field of view to measure a group of upper limb muscles^23^ and does not show detailed information of each forearm muscle. MRI can provide high contrast between different tissue and offer a large field of view, showing impressive advantages in measuring muscle volume. Through MRI, researchers can accurately identify muscles and easily select region of interest (ROI) manually. Considering that most congenital PRUS patients are young, an MRI scan without radiation is more acceptable and safer than CT.

Meanwhile, there is almost no report about preoperative biceps brachii muscle dysfunction or absence currently. Therefore, this study focused on correlating supinator ratio and radiographic characteristics among congenital PRUS patients. Muscle volume may vary with age, sex and activity level of the patient. To avoid bias caused by the above differences, the ratio of the area of peripheral muscle to supinator in the same MRI slice was defined as the indicator to supinator volume instead of merely measuring the surface area of supinator in this study. This retrospective study compared the MRI measured supinator muscle volume with a radiographic deformity in congenital PRUS, aiming to develop a new classification system to show their correlation and provide a more comprehensive understanding of congenital PRUS.

## Materials and methods

From January 2019 to October 2020, all patients diagnosed with congenital PRUS in two geographically separated pediatric Orthopedic centers were included in this research. The inclusion criteria were as follows: patients diagnosed with unilateral or bilateral congenital PRUS; without any other congenital malformation on the upper limb; never underwent surgery involving the upper limb. The exclusion criteria were: patients with any other congenital malformation on the upper limb or did not agree to undergo an MRI evaluation. The study was approved by the Ethics Committee of Tongji Medical College, Huazhong University of Science and Technology (IORG No: IORG0003571). All methods were performed in accordance with the relevant guidelines and regulations. The patient's legal guardians signed informed consent after they were informed about the purpose and procedure of the study, and images might be used for medical research.

Bilateral forearm radiographic images were collected from each patient once they were diagnosed with congenital PRUS according to Tachdjian^17,24^ and Cleary & Omer^18^ (Table 1) classification system by one radiologist and an orthopaedic surgeon. The radiographic characteristics of each forearm were recorded (Table 2).

**Table 1:** Radiographic manifestation of cleary & omer classification.

Type	Manifestation
I	Fibrous ankylosis with normal radial head
II	Osseous synostosis with normal radial head
III	Osseous synostosis with posteriorly dislocated and hypoplastic radial head
IV	Pseudo-synostosis and anteriorly dislocated, mushroom-shaped radial head

**Table 2:** Demographics information and imaging data for included cases.

No	Age	Gender	Left			Right		
			Cleary classification	Tachdjian's classification	Ratio	Cleary classification	Tachdjian's classification	Ratio
1	7	Male	3	2	5.845	3	2	6.257
2	3	Female	2	2	4.339	2	2	4.351
3	1	Male	3	2	3.176	1	Untyped	2.542
4	6	Male	4	Untyped	2.499	4	Untyped	2.068
5	2	Male	1	Untyped	2.036	Untyped	1	9.768
6	2	Male	Normal	Normal	1.201	Untyped	1	10.651
7	4	Male	3	2	6.857	Untyped	1	11.412
8	4	Male	Untyped	1	13.820	3	2	6.097
9	4	Female	2	2	4.306	Normal	Normal	1.324
10	3	Male	1	Untyped	2.986	3	2	3.700
11	2	Female	3	2	4.103	3	2	4.027
12	10	Male	3	2	5.55	Normal	Normal	1.23
13	2	Male	3	2	6.208	Normal	Normal	2.427
14	5	Male	1	Untyped	3.01	Untyped	1	8.155
15	8	Male	Untyped	1	5.784	Untyped	1	7.718
16	3	Female	Normal	Normal	2.485	3	2	4.257

**Figure 1:**
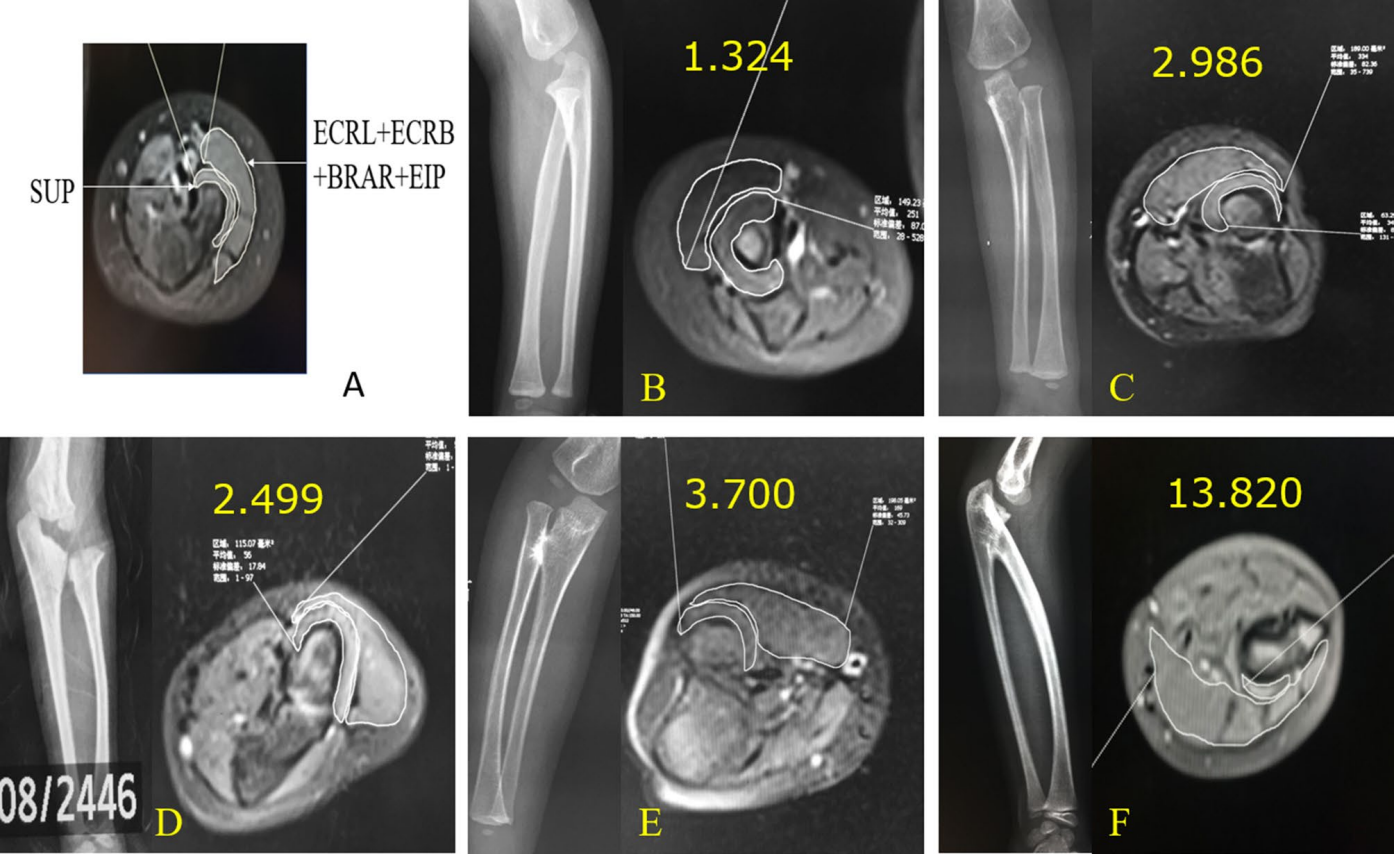
(**A**) The muscle selection of (ECRL + ECRB + BRAR + EIP) and supinator. (**B**) Radiography and MR image of the normal forearm with a supinator ratio of 1.324. (**C**) X-ray, MR image and supinator ratio of MRI & X-ray type I forearm, also classified as Cleary & Omer type I: Fibrous pseudo-synostosis with any shape of the radial head. (**D**) Radiography, MR image and supinator ratio of MRI & X-ray type I forearm, also classified as Cleary & Omer type IV. (**E**) Radiography, MR image and supinator ratio of MRI & X-ray type II: radiographic osseous synostosis with radial head dislocated. (**F**) Radiography, MR image and supinator ratio of MRI & X-ray type III, Tachdjian type I: radiographic radial head unobservable and osseous synostosis with the ulna.

MRI of both forearms (affected and normal or affected contralateral) was performed in a neutral forearm position with a 3.0 T MR imaging system (Discovery 750 W GE health care). The MRI of five children (younger than 3 years) were performed under sedation with chloral hydrate. The transverse MRI images measurement at the most proximal slice where the cartilage of the radial head could be observed. Considering that the cartilage of the radial head is unobservable in some congenital PRUS cases, the measurement slice was defined in these cases as the most proximal slice where the radius abnormally widened. All the MR image continuous acquisitions were 4.0 mm in thickness. The epimysia of muscles were traced manually, and the areas of the ROI were performed with commercial workstations (GE, ADW 4.6). The MRI was evaluated by an experienced radiologist. After that, an orthopaedic surgeon re-examined the scans and ensured the ROI selection consistent among all cases. In the starting slice of each forearm, the area of extensor carpi radialis longus, extensor carpi radialis brevis, brachioradialis muscle and extensor indicis muscle (referred to as peripheral muscle in the following) were measured as one ROI. At the same time, the area of the ipsilateral supinator was measured as another. The ratio of the surface area of peripheral muscle to supinator (named supinator ratio) in each forearm was calculated and regarded as an indicator of the volume of supinator (Fig. 1a). The supinator ratio of each forearm was reviewed again, combining the radiographic characteristics.

One-way ANOVA test, Student’s t test and SNK-q test were conducted using Rstudio (Boston, MA). All supinator ratios were shown as median [25th percentile, 75th percentile].

## Results

Table 2 shows the general information of the patients. Nineteen patients diagnosed with congenital PRUS were identified. Three of them did not agree to undergo an MRI evaluation, so they were excluded from the study. Twenty-seven congenital PRUS forearms of sixteen patients were included in this study. Eleven patients had bilateral involvement. Out of 16 patients, 12 were male, and 4 were female, with an average of 4.1 years (range 1–10 years). Among the PRUS, 14 were on the left side, and 13 were on the right side.

**Figure 2:**
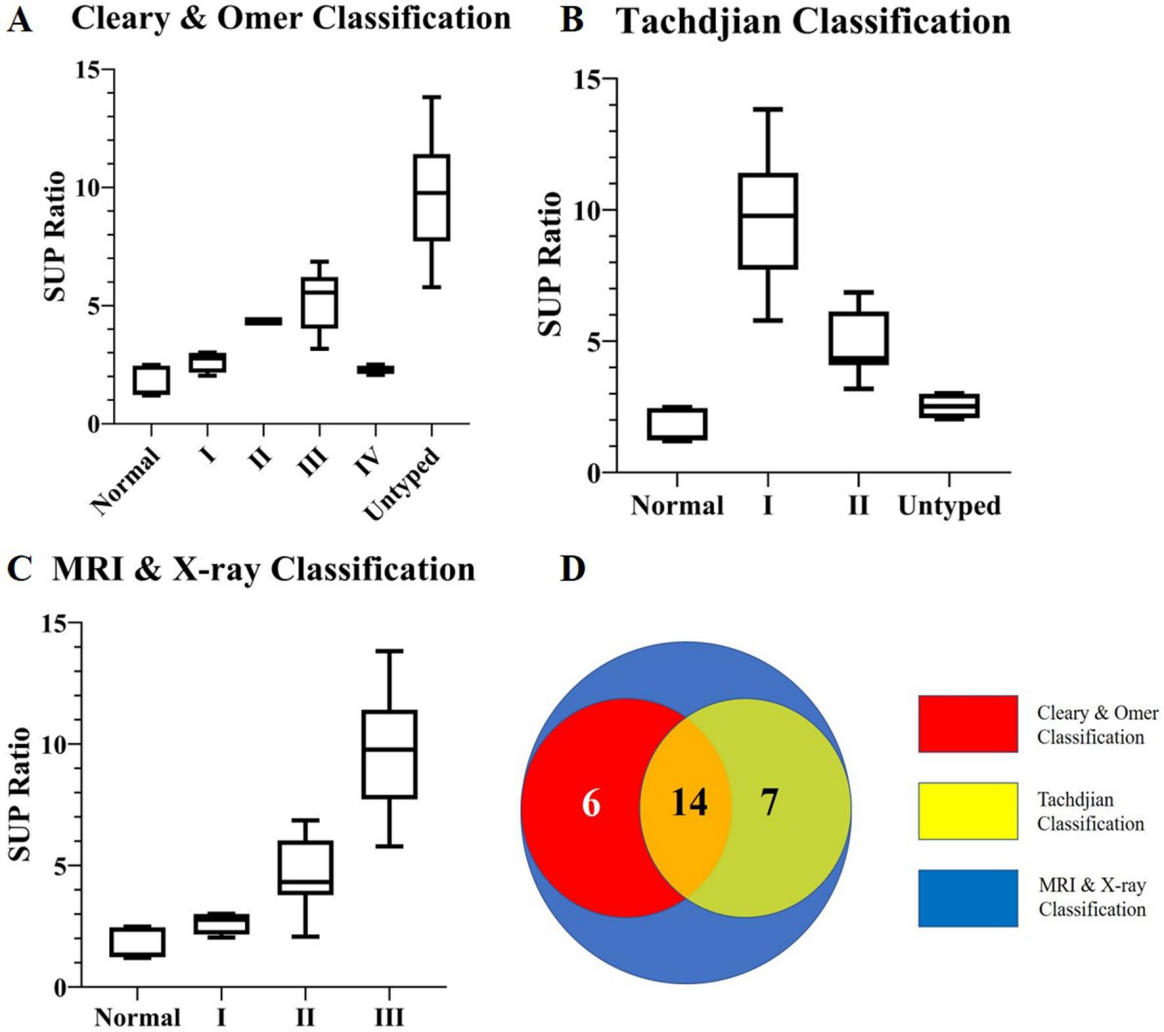
The distribution of SUP ratio of forearms included in this study in three classifications (Cleary & Omer (**A**), Tachdjian (**B**) and MRI & X-ray (**C**)) and the relationship of coverage between the three classifications (**D**). Both Cleary & Omer classification and Tachdjian classification could neither cover all congenital PRUS forearms in this study nor make supinator ratio follow the ladder-shaped distribution. MRI & X-ray classification covered all forearms and provided ladder-shaped distribution of supinator ratio between type I, II, III.

According to radiographic characteristics, 7 forearms had osseous synostosis with radial head unobservable and classified as Tachdjian type I, which were unable to classify by Cleary & Omer. Fourteen forearms were classified as Tachdjian type II, 3 were classified as Cleary & Omer type II PRUS, and the other 11 were classified as Cleary & Omer type III. Moreover, 4 forearms had fibrous pseudo-synostosis, and 2 had synostosis with anteriorly dislocated mushroom-shaped radial head. However, these were not included in the Tachdjian classification system but classified as Cleary & Omer type I and type IV, respectively. The distribution of these forearms is shown in Table 2 and Fig. 2d.

The supinator ratios were 9.8 [7.9, 11.0] for Tachdjian type I and 4.4 [4.1, 6.0] for Tachdjian type II. For Cleary & Omer classification, the supinator ratios were 2.8 [2.4, 3.0], 4.3 [4.3, 4.6], 5.6 [4.1, 6.2], 2.3 [2.2, 2.4] for type I, II, III, IV, respectively. The supinator ratios and classifications of each forearm are shown in Table 3. There was no significant difference of the SUP ratio among Cleary & Omer classification type I, II, III and IV. The SUP ratios of Tachdjian’s type I cases were significantly higher than Tachdjian’s type II cases.

**Table 3:** Comparison between Tachdjian’s, Cleary & Omer, MRI & X-ray Based classification systems.

Classification	Fusion	Radial Head	SUP Ratio
Normal	NA	NA	1.32[1.23,2.43]
MRI & X-ray type I	Fibrous	Any	2.76[2.42,2.99]
MRI & X-ray type II	Osseous	Normal or dislocated	4.32[3.95,5.91]
MRI & X-ray type III	Osseous	Unobservable	9.77[7.94,11.03]
Tachdjian's type I	Osseous	Unobservable	9.77[7.94,11.03]
Tachdjian's type II	Fibrous or Osseous	Dislocated	4.35[4.14,6.03]
Cleary & Omer type I	Fibrous	Normal	2.76[2.42,2.99]
Cleary & Omer type II	Osseous	Normal	4.34[4.32,4.35]
Cleary & Omer type III	Osseous	Posteriorly dislocated and hypoplastic	5.55[4.07,6.15]
Cleary & Omer type IV	Pseudo-synostosis	Anteriorly dislocated and mushroom-shaped	2.28[2.18,2.39]

**Figure 3:**
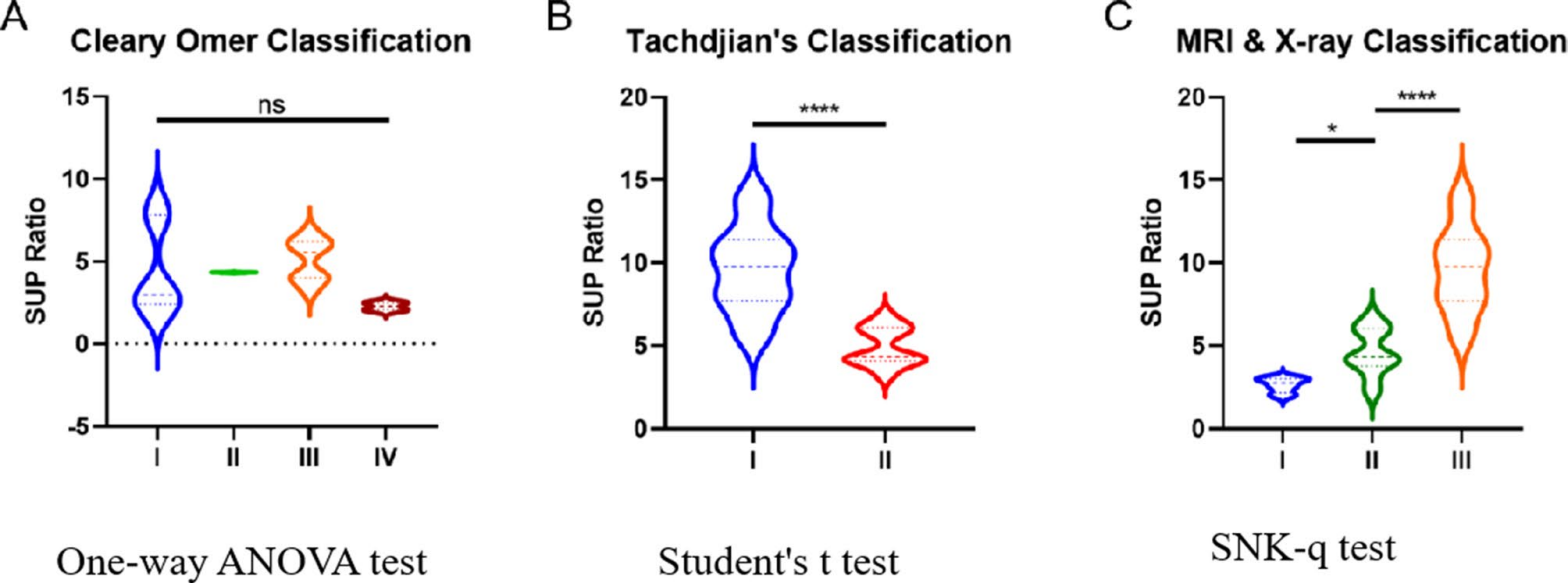
(**A**) There was no significant difference of the SUP ratio among each type of Cleary & Omer cases. (**B**) The SUP ratios of Tachdjian’s type I cases were significantly higher than the Tachdjian’s type II cases. (**C**) Significant differences of SUP ratio were found between MRI & X-ray type I, II, and III cases.

Therefore, a new classification system was proposed using MRI & X-ray, mainly based on MRI measured supinator ratios and radiographic fibrous pseudo-synostosis or osseous synostosis characteristics (Table 4). Type I: radiographic fibrous pseudo-synostosis with any shape of the radial head; type II: radiographic osseous synostosis with or without the dislocated radial head; type III: radiographic radial head unobservable and osseous synostosis with ulna (Fig. 1). The distribution of supinator ratios also supports this new classification. (shown in Fig. 2c) The SUP ratio between MRI & X-ray type I, II, and III cases show significant differences (Fig. 3). In this novel classification system, the supinator ratios were 2.8 [2.4,3.0], 4.3 [4.0,5.9], 9.8 [7.9,11.0] for type I, II, III, respectively. And, the supinator ratios for normal forearms are 1.3 [1.2, 2.4].

**Table 4:** MRI & X-ray based congenital PRUS classification.

Type	Radiographic manifestation
I	Fibrous pseudo-synostosis with any shape of the radial head
II	Osseous synostosis with or without dislocated radial head
III	Radiographic radial head unobservable and osseous synostosis with the ulna

## Discussion

In this study, the supinator ratios in forearms with true osseous synostosis and unobservable radial head (9.8, 7.9 ~ 11.0) were higher than those in other forearms, classified as Tachdjian type I and was not included in Cleary & Omer classification (Table 3). Supinator ratios of forearms with true osseous synostosis were smaller than those of Tachdjian type I forearms but larger than ratios of forearms with fibrous pseudo-synostosis. These could be interpreted as poor development of supinator in forearms with osseous synostosis and unobservable radial head. However, none of the previous reports, except Kao et al.'s hypothesis that the supinator might not exist, has reported the complete absence of supinator muscle. The results also confirmed that the supinator muscles might be atrophied or poorly developed but never be entirely absent^21^.

Cleary & Omer type I and type IV forearms showed similar supinator ratios. The association between Cleary & Omer type II and type III forearms is identical. Elliot et al.^4^ proposed that osseous synostosis and posteriorly dislocation of the radial head were the different radiographic manifestations of the same development abnormality. In contrast, anterior dislocation of the radial head resulted from abnormal external forces during the late fetal period or even postnatal period. No significant difference of the SUP ratio between Cleary & Omer classification type II and III, or between type I and IV were found. However, Cleary & Omer classification type I and IV show similar radiographic fibrous pseudo-synostosis characteristics, while type II and III similar radiographic osseous synostosis characteristics. Therefore, Cleary & Omer type I and IV could be classified as MRI & X-ray type I while Cleary & Omer type II and type III could be classified as MRI & X-ray type II,.

There is no consensus on treatment selection and surgical indications for congenital PRUS. Several surgical techniques have been described, but the outcomes are still uncertain^3,8,17,21,25–31^. Complications such as recurrence of synostosis, insufficient supination, compartment syndrome, nerve palsy, and vascular injuries were reported^3,10,32,33^. As for indications for surgery, some authors^9,12,14^ proposed that conducting surgery depends on both subjective symptoms and bony deformity, while Hwang et al.^13^ considered the patient's complaint to be more indicative for surgery. Moreover, Simmons et al.^6^ believed that patients with pronation larger than 60° must undergo surgical intervention. Existing classification systems, such as Cleary & Omer classification and Tachdjian classification, do not cover all congenital PRUS cases reported in clinical practice^10,17^. Cleary & Omer classification system fails to cover issues with true osseous synostosis and unobservable radial head, while Tachdjian classification does not include congenital PRUS forearms with fibrous pseudo-synostosis with the normal radial head (Fig. 2). Both classification systems merely considered radiographic characteristics, ignoring the status of forearm muscles and application in the management is largely limited^9^. Meanwhile, this new classification strategy considers osseous deformity and muscle volume simultaneously, providing a more comprehensive understanding of this rare deformity. Therefore, this study might provide another perspective on preoperative assessment. If a surgical intervention was taken into consideration for a PRUS patient, MRI & X-ray type II should has priority to type I, and MRI & X-ray type III should has priority to type II because of the poor development of supinator in forearms which might lead to poor functional prognosis.

Although the clinical recommendation from supinator ratio, this novel classification still require further research, and it reminds surgeons about the possible impact of supinator muscle developmental status on prognosis. The correlation between supinator muscle volume and pronation deformity degree in this study was not significant, which might be the result of individual differences as well as the limitation of sample size. There are various limitations to this study. The limited sample size could not offer more reference to the range of supinator ratio of each type of congenital PRUS forearms. Because the average value of supinator ratio in healthy children correlating with age is still unknown, it would be meaningful to have a healthy population and look for an average supinator ratio in these patients correlating with age in further research. Due to the deformity, the forearms position might not be entirely consistent during MRI measurement. There is no doubt that the comparison between ratios of bilateral forearms among which one side is normal will be more convincing. It can vastly diminish errors induced by individual differences. But it is unpractical when the patient's bilateral forearms are all deformed. The inter-and intra-observer variability needs to be validated in further research because only one radiologist and an orthopaedic surgeon performed the ROI selection of MRI measurement in this study. A further prospective study with more cases is required to obtain a more specific relationship between supinator development and radiographic characteristics of congenital PRUS. A linear relationship is preferred. The accuracy of MRI-based muscle volume measurement remains further confirmation because currently published studies still vary in the performance of error control. Tingart et al.^34^ proposed a relatively precise rotator cuff muscle volume measurement, with a variability of less than 4%. Eng et al.^23^ also practised this method, but more considerable variability was reported (10%). More cases and further study are required to establish a more reliable MRI based muscle volume measurement. Further, there is still a long way to go for this novel classification to provide a practical clinical recommendation for the treatment and prognosis of congenital PRUS. This study is meant for the perspective of muscles' contribution and comprehensive coverage of radiographic deformity that make the findings from this study different. Further research concerning postoperative long-term changes of supination function among congenital PRUS patients with varying ratios of supinator is required to establish more practical advice. This novel classification and supinator ratios might help surgeons and patients judge whether and when to conduct surgical treatment in the future.

## Conclusions

This retrospective study revealed the relation between MRI measured supinator muscle volume and radiographic deformity of congenital PRUS. Supinator muscles were observed in all congenital PRUS cases. A novel classification was proposed, providing a more comprehensive understanding of congenital PRUS and.

## Data Availability

The datasets generated during and/or analyzed during the current study are available from the corresponding author on reasonable request.
